# Hard X-ray inverse Compton scattering at photon energy of 87.5 keV

**DOI:** 10.1038/s41598-024-68170-8

**Published:** 2024-08-09

**Authors:** Yusuke Sakai, Marcus Babzien, Mikhail Fedurin, Karl Kusche, Oliver Williams, Atsushi Fukasawa, Brian Naranjo, Alex Murokh, Ronald Agustsson, Andrew Simmonds, Paul Jacob, George Stenby, Robert Malone, Mikhail Polyanskiy, Igor Pogorelsky, Mark Palmer, James Rosenzweig

**Affiliations:** 1 University of California at Los Angeles, Los Angeles, CA 90095 USA; 2 RadiaBeam Technologies, LLC, Los Angeles, CA 90404 USA; 3 Brookhaven National Laboratory, Upton, NY 11973 USA

**Keywords:** Biological techniques, Biophysics, Biotechnology, Cancer, Cell biology, Health care, Medical research, Oncology, Energy science and technology, Engineering, Materials science, Mathematics and computing, Optics and photonics, Physics

## Abstract

Production of hard X-ray via inverse Compton scattering at photon energies below 100 keV range aimed at potential applications in medicine and material research is reported. Experiments have been performed at the Brookhaven National Laboratory, Accelerator Test Facility, employing the counter collision of a 70 MeV, 0.3 nC electron beam with a near infra-red Nd: YAG laser (1064 nm wavelength) pulse containing ~ 100 mJ in a single shot basis. The radiation distribution of the scattered photon beam is assessed to be sufficiently quasi monochromatic to produce clear contrast from the Au *K*- edge at 80.7 keV.

## Introduction

Feasibility studies on the development of an Inverse Compton Scattering (ICS)^1,2^ source enabled by counter collision of intense laser and bright electron beams have made considerable progress in recent years. Experiments have demonstrated the physics of linear and nonlinear ICS that enable applications in both the hard X-ray^3–8^ and gamma-ray regimes^9–11^. Synchrotron-radiation-based 3rd–4th generation light sources, such as high average flux synchrotron rings^12^ and pulsed coherent X-ray Free Electron Lasers (XFELs)^13^ at photon energies up to mid-hard X-rays (~  20 keV) are under active use. Meanwhile, demands for higher energy monochromatic photon sources, in the photon energy of sub 100 keV range, in bulk state material research^14,15^, medical^16^, and ultimately nuclear photonics research^9–11^ are increasing.

This provides the motivation for the actual applications of compact pulsed quasi monochromatic inverse Compton sources, where the so-called “laser undulator” operates at a micron period^2^, as opposed to cm period magnetic undulators. ICS enables production of much higher energy photons, where one may extend the reach of the source to over 100 keV, and on up to MeV or even GeV levels at modest electron beam energy. Compelling examples of potential application of monochromatic ICS X-ray sources in the service of medicine are X-ray computed tomographies^17^ and photon activation therapy. As an example, quasi monochromatic Compton source seems to be suitable for dose enhancement of photon activation therapy owing to *K* absorption edge utilizing gold nanoparticles examined in this report^16,18,19^.

### Energy efficiency of Compton scattering

The industrial adoption of compact ICS sources requires technical breakthroughs, particularly to enable operation at the repetition rate needed to overcome limitations on the number of incoherent scattered photons obtained per pulse. This is a fundamental issue, as ICS production is proportional to the Thomson scattering cross-section of laser quanta from an electron. Examples of proof-of-demonstration to maximize the average ICS photon flux has been reported, using methods such as energy recovery linacs (ERLs)^7^, and recirculated lasers in tandem with an electron bunch train configuration^20^ produced by the accelerator. Herein this report, emphasis on maximizing a X-ray yield per pulse and single shot measurement are described.

As the scattered photon energy increases, the energy efficiency increases proportionally, since recoil of an electron, i.e. the direct energy lost to the photon, becomes comparable to initial kinetic energy of an electron beam. For example, the counter collision of Nd: YAG laser with an 100 MeV electron boost near infrared laser of photon energy 1 eV to,

1 $$\hslash \frac{{2\omega }_{L}}{1-v/c}\approx4 {\gamma }^{2}\hslash {\omega }_{L}\sim 100 \text{keV}$$

by the relativistic Doppler blue-shift^1^. Here, *v* is e-beam velocity, *c* is the speed of light, *ω*_*L*_ is angular frequency of colliding laser, and *γ* is the relativistic Lorenz factor. This energy, defined as the Compton edge, approaches 0.1% of an electron beam’s initial kinetic energy. The total radiant energy contained by photon number of 10^9^ at photon energy of 100 keV corresponds to 16 µJ.

To further examine this example, if a near infrared laser of total energy 10 J is focused to a spot size of 10 µm, the areal number density of the photons becomes 10^24^ photons/cm^2^. As the Thomson cross-section is *σ*_*T*_ = 6.65 × 10^–25^ cm^2^, scattering of one photon per an electron can be anticipated^21^. Thus, if all of the 10^9^ electrons of an electron beam with 0.16 nC are involved in the interaction, the hard X-ray yield reaches 10^9^ per pulse in pico second timescale. Under these conditions, the efficiency of electron to photon transfer is ~ 0.1%. Since the majority of the radiant energy from relativistic electron beams is confined within an angular cone of ~ 1/*γ* < 10 mrad, in the ICS source we are discussing flux illuminating a target will be contained into a few cm^2^ size if the target is located a few meters away from the Compton interaction point (IP)^1^. Therefore, if the radiation deposition depth is 1–10 cm, 1000–10,000 shots using the irradiation of the ICS X-rays having 1 µJ per pulse allows deposition of ~ 1 mJ/cm^3^ ~ 1 mJ/g, for the case of water, to the target material that is equivalent to a dose of 1 Gy per volume. It should be noted that a Compton source does not maintain its spectral brightness at photon energies much above hard X-ray range, particularly when recoil of the scattered X-ray photon starts to be significant. Nevertheless, the advantages of pulsed controllability and directionality are unique to ICS sources as the photon energy enters the gamma ray regime.

### The Compton source spectrum

Regarding on the monochromaticity and spectral brightness of the Compton source, the features of the scattered radiation include off-axis redshifting due to the relative angle of the observation with respect to the electron direction, and the on-axis redshift due to the nonlinear Compton interaction by relativistically intense laser. These two effects together are written by^12,21^,

2 $${\omega }_{\text{ICS}}\approx \frac{4{\gamma }^{2}{\omega }_{L}}{1+{\gamma }^{2}{\Theta }^{2}+{{a}_{0}}^{2}/2} .$$

Here *Θ* is the radiation angle with respect to the electron-beam axis, the central direction of average Poynting vector of fundamental radiation, and *a*_0_ is a normalized vector potential of the laser described in the next.

#### Off-axis redshift effect

Off-axis redshift associated with the relative angle of the observation and propagation direction of electron beam, at radiation angle of *Θ* = 1/*γ*, photon energy is off-axis redshifted to one-half that of the Compton edge according to Eq. (2). The effective area of radiation target can be estimated by assuming the distance from the Compton IP to the target to be a few meters, and cylindrical shaped target with diameter of 1 cm, acceptance of radiation angle becomes a few mrad. From Eq. (2), the photon energy at *Θ* = 1 mrad corresponds, 1/[1 + (*γΘ*)^2^] = 1/[1 + (70 MeV/0.51 MeV × 1 mrad)^2^] ~ 0.99.

#### Redshift due to intense laser effect

**Figure 1:**
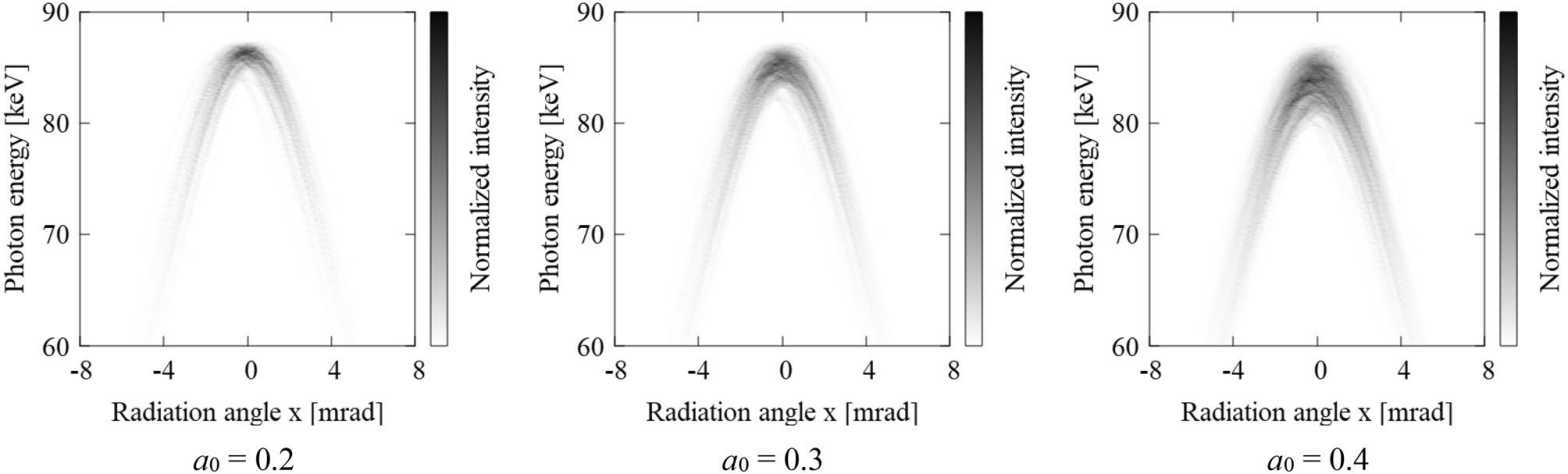
Double differential spectrum of inverse Compton scattering for Gaussian beams. Electron beam energy: 70 MeV, Laser wavelength: 1064 nm, Normalized emittance *ε:* 2 mm mrad, e-beam and laser transverse sizes are equal: *σ*_*e, x, y*_ = *σ*_*L, x, y*_: 30 µm, Normalized vector potential of laser: *a*_0_: 0.2, 0.3, 0.4 from left to right respectively.

Figure 1 shows the numerically calculated double-differential spectrums (DDS) of an ICS source based on relativistic Lienard–Wiechert (LW) potential formalism with temporal and spatial Gaussian beam distributions. The electron beam energy is 70 MeV, while the normalized emittance is *ε* = 2 mm mrad and beam sizes are *σ*_*e*, *x*, *y*_ = σ_*L*, *x*, *y*_ = 30 µm. As the Lorenz factor *γ* = 137, the angle of the radiation cone is 1/*γ* = 7.3 mrad, and Compton edge is 87.5 keV. The nonlinear effect due to the amplitude of the laser field, where the normalized vector potential of the laser *a*_0_ = 0.2, 0.3, 0.4 are indicated in Fig. 1. Asymmetry in the DDS is due to a random number utilized for initial Guassian distributions of electron macro particles. Here *a*_0_, is defined as the peak vector potential of the laser wave, normalized to ${m}_{e}c$,

3 $${a}_{0}\equiv e{E}_{0}\,\left({\lambda }_{{L}}/2\pi \right)/{m}_{\text{e}}{c}^{2},$$

where *E*_0_ is the peak electric field amplitude of a laser that is linearly polarized in the *x* direction, and *λ*_*L*_ is the laser wavelength. If *a*_0_ approaches unity, the transverse motion of the electron takes on relativistic character, tracing a "*figure-eight*" motion. Or, when the electron transverse velocity *v*_*x*_ approaches light speed, the magnetic component of the Lorentz force *B*_*y*_ × *v*_*x*_/*c* becomes non-negligible. Since an electron obtains the equivalent momentum equal to or exceeding ${m}_{e}c$ in one laser cycle, the electron beam slows down according to the observer in the lab, resulting in an on-axis redshifting of the radiation spectrum.

Maximizing the laser intensity is an obvious and straightforward way to increase X-ray flux in order to minimize operational electron-beam time to keep the high energy radiation in the environment, and to attain the desired doses. However, the factor limiting the utility of this approach is the spectral redshifting. As the laser intensity increases, the spectrum broadens and the X-ray bandwidth grows proportionately up ~ 10% for *a*_0_ = 0.4. Detailed experimental studies of the intense laser effect by utilizing a long wavelength terawatt (TW) CO_2_ laser has been reported as an extreme case for *a*_0_ > 0.5^22,23^. Note that *a*_0_ is proportional to square root of laser intensity *I*_*L*_ ~ *E*_0_^2^. In Fig. 1, the spectrum shows broadening instead of simple redshifting due to assumed temporal and spatial Gaussian distributions of laser and electron beams.

In this experiment, a Nd:YAG laser (at wavelength 1064 nm) having pulse duration of ~ 15 ps FWHM with bandwidth near transform-limited and beam quality factor *M*_2_ < 2.0. The laser energy per shot is typically 100 mJ, and thus peak power of the laser is 6.3 GW. Assuming the spot the size of the laser at the interaction point is ~ 20–30 μm, the peak intensity of ~ 5.5 × 10^9^ [W/m^2^], which corresponds to an electric field ~ 2 × 10^11^ [V/m]. Thus, the estimated normalized vector potential is *a*_0_~ 0.066 ~ 0.1. Then, the nonlinear redshift of the photon energy is estimated to be less than 1%.

#### Emittance and focusing of e-beam effect

A redshift effect associated with emittance *ε* and size of electron-beam is evaluated through transverse momentum *Δp*_*x,y*_ at the IP^21^. In Eq. (2), the radiation angle *Θ* can be replaced by relative angle of electron’s propagation direction to the observation screen due to the non-negligible transverse electron momentum induced by tight focusing,

4 $${\gamma }^{2}{\Theta }^{2}\leftrightarrow {\gamma }^{2}{\left(\Delta {p}_{x,y}/{p}_{z}\right)}^{2}.$$

As an example, with the parameters of the current experiment at BNL, an electron-beam radius *σ*_*x,y*_ ∼ 30 µm corresponds to beta function (effective longitudinal length at the transverse beam size) of *β* ∼ 6 cm at 70 MeV electron beam energy, as given by the emittance^21^,

5 $$\epsilon/\gamma = \sigma\sigma' = \sigma^{2}/\beta.$$

Hence, the electron beam angular divergence is approximately *σ*` ~ *σ*/*β* ~ Δ*p*_*x,y*_/*p*_*z*_ = 0.5 mrad. The redshifting due to the emittance effect becomes 1/[1 + (*γ*_0_ × Δ*p*_*x,y*_/*p*_*z*_)^2^] < 0.99.

If the electron beam size is focused further to the *σ*_x,y_ ≤ 10 µm range, with beta function shortened to *β* ~ mm, divergence of an electron increases to *σ*` > 1 mrad, resulting in a several % broadening of X-ray spectrum. Although tight focus of e-beam indeed increases scattering probability to optimize X-ray flux, depending on applications it is necessary to consider the spectral brightness. Alternatively, as the e-beam angle can be expressed *σ*` ~ *ε*/(*γσ),* lowering emittance could be a robust method to further improve the efficiency and spectral quality of the source.

**Figure 2:**
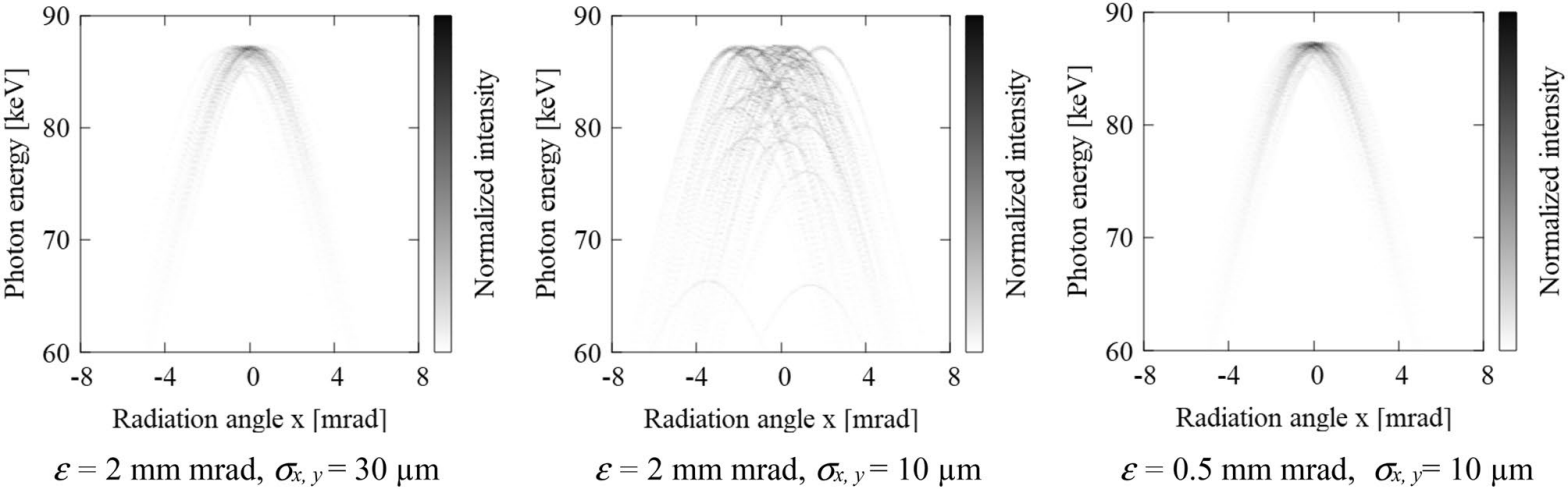
Numerically calculated double differential spectrum of hard X-ray Compton source with various e-beam emittance and focusing size at laser intensity of *a*_0_ = 0.1. Left: *ε* = 2 mm mrad, *σ*_*x, y*_ = 30 µm, Middle: *ε* = 2 mm mrad, *σ*_*x, y*_ = 10 µm, Left: *ε* = 0.5 mm mrad, *σ*_*x, y*_ = 10 µm.

In this context, cryogenic high gradient photo-electron RF gun research and development is actively under investigation. Here the peak brightness of Thomson scattering is proportional to ~ 1/*σ*_*e,x,y*_^2^ ~ 1/*ε*^2^, if the spot size of the laser is fixed to be smaller than that of e-beam and divergence of the e-beam *σ*` to be constant. As the emittance of an electron beam at a low energy space charge dominated region is approximately proportional to 1/*γ*^2^, maximizing the accelerating field of an electron gun by reducing breakdown probability at lower temperature is considered to be an effective solution^24,25^. Figure 2 shows examples of the numerical calculation of the DDS as a function of the emittance and electron-beam size *σ*_*e,x,y*_. If the beam with emittance of 2 mm-mrad is focused to sub-10 μm range, it is apparent that the spectrum broadening could significantly exceed redshifting due to the intense laser field for the case of *a*_0_ = 0.1, as shown in Fig. 2 (middle). However, if the emittance could be lowered to 0.5 mm mrad at *σ*_*e,x,y*_ ~ 10 μm, the X-ray band width again becomes below < 1% as shown in Fig. 2 (right).

The characteristics of a sub-10 keV ICS X-ray radiation in quasi linear regime have been previously investigated by utilizing a CO_2_ laser at wavelength of 10.6 μm in BNL-ATF, that was performed by using a Fe *K*-edge filter^26^. In the current experiment, by utilizing the Nd: YAG laser at a wavelength of 1064 nm, emission of monochromatic hard X-ray at photon energy of sub-100 keV, in a linear regime, has been similarly examined based on the characteristic of radiation distribution observed on a screen located downstream of a Au *K*-edge filter.

## Experimental results

**Figure 3:**
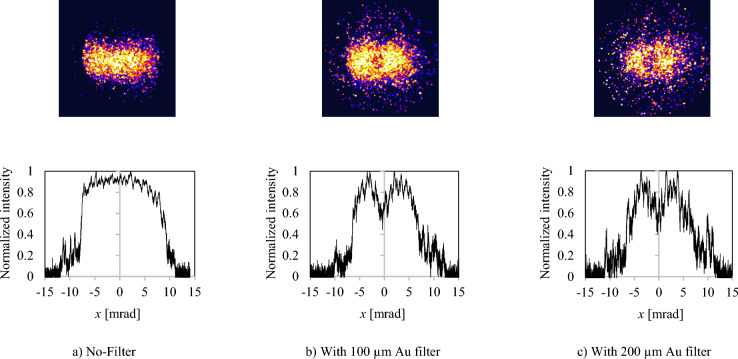
Experimentally detected ICS hard X-ray in a single shot on a MCP screen at photon energy of 87.5 keV through (**a**) No-Filter, (**b**) With 100 µm Au filter, (**c**) With 200 µm Au filter in BNL-ATF. e-beam energy: 70 MeV, Wavelength of Nd YAG laser: 1064 nm. The contrast is normalized to each data for qualitative comparison. Background without e-beam subtracted. Bottom plot corresponds to line intensity profiles at *y* = 0 axis.

The experimental conditions in the measurements reported here correspond to those used in the numerical calculations shown in Fig. 2, left. The electron beam energy is 70 MeV, while the normalized emittance is *ε* = 2 mm mrad and beam sizes are *σ*_*e, x, y*_ ∼ *σ*_*L, x, y*_ ∼ 30 µm. Figure 3 shows the experimentally observed attenuation of the single shot ICS photons produced in the ICS interaction using Au filters (or their absence): (a) no filter; (b) with 100 µm Au filter; and (c) with 200 µm Au filter. In standard e-beam operation of BNL-ATF, the bunch number is 1, pulse width is *σ*_*t*_ ~ 3 ps with repetition rate 2 Hz. Energy band width of an electron beam is approximately 0.1%, and center beam energy is tunable between 50 and 80 MeV in S band linac section without affecting emittance.

**Figure 4:**
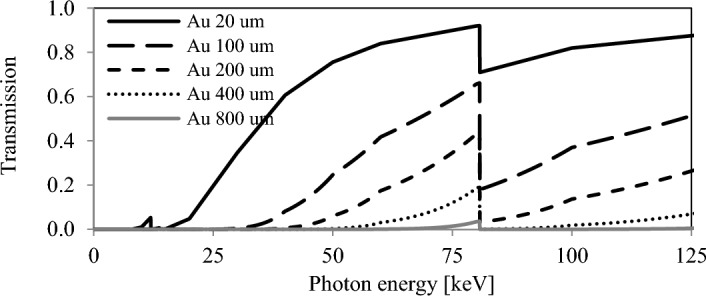
Spectral transmission of Au filters.

Bottom plot profile corresponds to line intensity profiles at *y* = 0 axis. The X-rays were detected by a micro-channel plate (MCP) as an observation screen. Transmission curves of X-rays through these filters are shown in Fig. 4. A Au 100 µm thick filter attenuates the majority of the on-axis components inside an opening angle of ~ 2 mrad.

As shown in the numerical DDS calculated (or according to Eq. (2)), the 80.7 keV Au *K*-edge should be located at an radiation angle of ~ 2 mrad. The increase of Au filter thickness to 200 µm attenuates the components further. Also, since the attenuation of below ~ 60 keV further increases, the off-axis radiation intensity decreases beyond ~ 5 mrad. In order to set optimum constant MCP bias for detection of hard X-rays through Au filters, the MCP image in the case of No-filter is saturated, resulting in the flat-top uniform distribution in this experiment. Meanwhile, for the case with Au filters, the radiation distributions are fully responsive to the X-ray intensity. The cutoff shown in a) no-filter case is caused by the beam pipe restriction. The quantum efficiency (QE) of MCP at lower energy is assumed to be much higher than higher energy components. Since available QE data, especially sub 100 keV range is lacking, only qualitative information has been obtained in this experiment.

## Discussion

**Figure 5:**
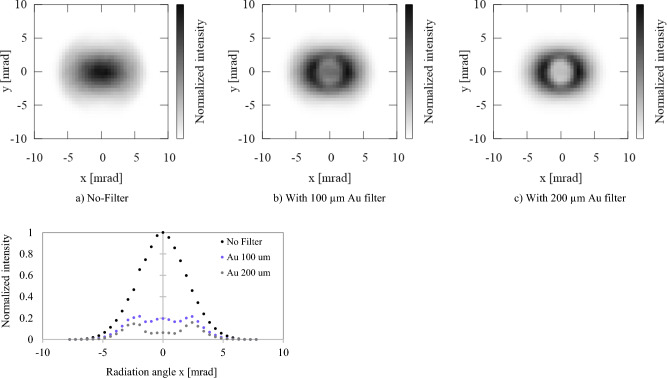
Numerically calculated attenuation of ICS hard X-ray at photon energy of 87.5 keV through (**a**) No-Filter, (**b**) With 100 µm Au filter, (**c**) With 200 µm Au filter. Normalized vector potential of laser *a*_0_: 0.1, with YAG lasera having *σ*_*L, x, y*_ = 30 μm. Normalized emittance *ε*: 2 mm mrad, e-beam transverse size σ_*e, x, y*_: 30 µm.

2D radiation intensity distributions produced via numerical calculation of the Lienard Wiechert potential with the experimental parameters used are shown in Fig. 5. Initial conditions of the electron beam are set to: normalized emittance *ε* = 2 mm mrad; electron-beam and laser transverse size at Compton IP: *σ*_*e, x, y*_ = *σ*_*L, x, y*_ = 30 µm; normalized vector potential is *a*_0_ = 0.1. The lack of cylindrical symmetry comes from the laser’s linear polarization in *x* direction. For the case with Au filters of thickness 100 μm and 200 μm, local peaks with an annular ring pattern are positioned at 2.5 mrad. Note that in Fig. 5, the 2-dimensional intensity distribution is normalized in each case, while plot profiles, integrated within 10% around *y* = 0 axis, are normalized to the case with no filter. The on-axis absorption of hard X-rays for Au filters of thickness 100 μm and 200 μm is calculated to be 80% and 90%, respectively. In addition, the ratio of the on-axis intensity to the annular local peak for each case is 0.8 and 0.4. Despite the limited resolution of observation in a single shot, the contrast of the radiation pattern is in agreement with the Lienard Wiechert potential numerical calculation for the experimental Gaussian beam parameters measured.

According to the above-mentioned laser parameter, the peak intensity of the Nd: YAG laser is estimated to be 5.5 × 10^9^ W/m^2^, resulting in an area number density of photons of 5 × 10^23^ ph/cm^2^. With Thomson scattering cross section *σ*_*T*_ = 6.65 × 10^–25^ cm^2^, one scattered photon per 100 electron is expected in this experiment. In addition, as the 2× Rayleigh length of the YAG laser pulse, on the order of sub mm, is shorter than beta-function of electron-beam, the effective interaction time is limited by the bunch length of electron beam, σ_*t*_ ~ 3 ps, which is notably shorter than the pulse length of laser, with FWHM (= 2.35 Sigma) ~ 15 ps. In this case, ≤ 50% of laser pulse can be involved in the interaction. Therefore, as the electron number per 300 pC is 1.9 × 10^9^, the estimated X-ray photon number yield is in the order of (0.01 photons) (1.9 × 10^9^) × (*σ*_*L, x, y*_ / *σ*_*e, x, y*_)^2^ × (0.5) ~ 10^6^ photons/pulse. In the experiment, the actual size of the electron beam and the laser at the center of the IP may fluctuate from *σ*_*e*, *x*, *y*_ = *σ*_*L*, *x*, *y*_ = 30 µm range. Especially, shape of an electron beam tends to be non-Gaussian for tight focusing, which normally lowers the actual X-ray yield. This number is consistent with an approximate measurement by utilizing a Si photodiode detector (Canberra, A300) based on an attenuation length of the photon energy compared to that of ~ 10 keV X-ray^22^. The details of quantitative photon number measurement and QE calibration of the MCP will be required.

### Considerations of potential applications

As an example of medical application with high-*Z* materials and ICS X-rays, we describe here an intuitive process of photon activation of Au nano-particles (AuNP). This application is directly based on the experimentally observed *K*-edge absorption of > 80 keV X-rays by Au filters.

The use of AuNP for photon activation^16,18,19,27,28^ relies on the higher optical thickness of high-*Z* metal nanoparticles and the relative transparency of surrounding water and organic materials. First, the inner *K*-shell electrons of high-*Z* matter-based particles behave as an initial absorber. Then, high energy electrons induced by first photoionization or secondary low energy Auger electrons^29^ created via subsequent autoionization^12^ escape from the surface of the particles. In the biomedical case, during the propagation of such electrons through surrounding water, excitation of free radicals such as hydroxide (OH) ions induce eventual photon-based activation and subsequent delivery on an enhanced dose^28^.

As a useful approximation, the integrated material density along an optical X-ray path can be studied through high energy photon scattering. With the equivalent material density of a 20 µm thick gold filter, no notable absorption is expected for 100 keV X-rays, as indicated in Fig. 4. The enhancement starts to be seen for Au thickness of 100 µm at photon energy around the *K*-absorption edge, as experimentally observed. For Au with thickness of 800 µm, the transmission essentially vanishes over the entire spectrum range. Assuming a typical Au NP has a square shape and that they are equally spaced, a density of 100 µm thick Au sheet in a cubic cm of water corresponds to 193 mg/g uptake, where the density of Au and H_2_O are 19.3 g/cm^3^ and 0.997 g/cm^3^ respectively. Approximately 80% of the hard X-rays are expected to be absorbed per 1 cm propagation in this case.

**Figure 6:**
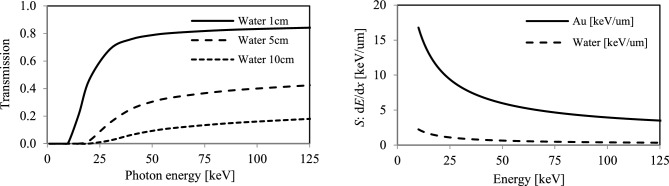
Left: Transmission curves of water. Right: Stopping power *S*(*E*_*e, free*_) as a function of kinetic energy *E*_*e, free*_ of free electron in units of keV per µm.

Figure 6 (left) shows the approximate X-ray transmission curves in water with thickness of 1 cm, 5 cm and 10 cm. For a volume of water with thickness of 1 cm and 5 cm, absorption at 80–90 keV X-ray is ~ 20% and ~ 60% respectively higher than that of the 100’s µm thick Au sheets. This results in a dose enhancement. However, absorption contrast of hard X-rays through 10’s of cm water becomes negligible. After *K*-shell absorption of hard X-rays in the Au NP, efficient escape of an excited electron from a nanoparticle must occur, which necessitates consideration of particle size of the high-*Z* materials used.

To give a simple estimate, the stopping power of free electrons in a unit of keV/µm is shown in Fig. 6 (right). For 10 keV electrons, the stopping power in Au is 10 keV/µm, therefore a particle with diameter in a range of < 100 nm is sufficient to permit escape. If direct photo-electrons emitted from *K*-shell excitation is considered to be relevant, (since ionization energy is approximately − 13.6 *Z*^2^/*n*^2^ ~ 85 keV for *Z* = 79), the required X-ray energy extends to 100 keV range in order to yield excess kinetic energy of ~ 10 keV. However, approximately, the absorption probability is decreased by a factor of ~ 2 at X-ray energy of 100 keV from ~ 85 keV as photo ionization cross section is proportional to

6 $$\sigma_{{{\text{Ph}},{\text{ Ion}}}} \sim (\hslash \omega _{K} /\hslash \omega )^{{3}}$$

where $\hslash$ *ω*_*K*_ is the photon energy of the *K*-edge. This suggests the importance of the quasi monochromatic X-ray having bandwidth of several %. This expression is valid assuming a hydrogen-like wave function, and operation near *K*-edge energy. In this context, > 90% of radiation energy should be left for subsequent secondary relaxation processes. In this regard, Auger electron emission from the outer shell is a dominant factor.

Indeed, Auger electrons near *K*-shell generate medium energy free electrons in accord with the *L*-edge and *M*-edge energies of 11.9–14.3 keV and 2.2–2.4 keV respectively. Thus, efficient escape of *M*-shell Auger electrons from Au NP requires particle diameter of < 10’s of nm range. We note that a 100 µm thick Au filter occupies 1% of the volume of a 1 cm thick water layer. If the diameters of the Au NP spheres are 10 nm and 100 nm, the distance between equally spaced nano-particles becomes ~ 1 µm and ~ 10 µm, respectively. Free electrons should be able to propagate for micrometer ranges and thus uniform activation is anticipated. Details of Monte Carlo simulations of gold nanoparticle dose enhancement of Inverse-Compton based monoenergetic photon beams have been investigated^18,19^, and these studies illuminate our discussion further. The experimental capability of creating a monochromatic hard X-ray source enables the precise physical study of complicated microscopic activation processes including fast pulse effects. Depending on the beam parameters discussed above, X-ray bandwidth control and collimation could enhance localization of the dose owing to methods of utilizing thick bent crystals suggested by Zhong et al.^30^, which is under active consideration. For material research or trace element analysis by hard X-ray radiography, although surrounding materials will show larger variety, as long as atomic number of elements are relatively low, a similar description would be valid.

## Methods

The experiment has been conducted at BNL ATF Beam Line 1 (BL1), which is fed by a S-band RF based electron linear accelerator with a room temperature photoinjector driven by a 4 th harmonic of the Nd: Glass laser at wavelength of 266 nm. The part of the Nd: Glass laser utilized as a photo cathode injection is transported through the Nd:YAG amplifier operating at wavelength of 1064 nm to the experimental area, where the preamplifier increases the laser energy up to the 10 mJ range. To enable the Compton scattering interaction, at the end of beam line 1, the laser energy is further amplified to the 100 mJ level by a two pass through amplifier with a Nd:YAG rod of diameter 1 inch (Amplitude-Continuum Inc.).

**Figure 7:**
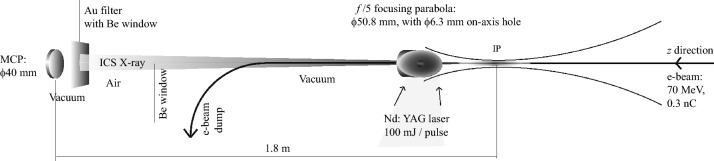
Schematic diagrams of Compton interaction optics. Nd: YAG laser is focused by *f*/5 OAP. OAP has on-axis holes of diameter 6.3 mm for the passage of a 70 MeV electron beam. The electron beam propagates along the *z* axis.

Schematic diagram of the setup is shown in Fig. 7. The laser is focused by a gold-coated off-axis parabola (OAP) with 50.8 mm diameter giving *f*/5 focusing. This mirror has a 6.3 mm diameter hole on-axis (Thorlabs MPD2109H6V6-M03-UA-SP series). The electron beam and the ICS X-rays travel in the *z*-direction through the on-axis hole, and a portion of incident YAG laser is picked off in the *y* direction as a timing reference. The Rayleigh length of the laser is measured to be < 500 μm by a longitudinal scan using a pinhole of diameter 100 μm. Both the electron beam and laser are spatially aligned through the pinhole located at the IP. Charge transmission of the e-beam though the pinhole is around 90 % as measured using a Faraday cup located in the end of the electron beam line, after passage of 90 deg dipole magnet. Timing within ps range is accomplished by the deflection of a near-infrared laser with an electron beam excited plasma in a 0.5 mm thick Si plate located at the Compton interaction point^31^.

A MCP of effective diameter 40 mm (PHOTONIS MCP40/12/10/8I60∶1EDRKBR6, P46), having a nominal center-to-center spacing of 12 µm and pore size of 10 µm, in combination with a phosphor screen^22^ and a CMOS camera (Basler Ace acA4112-20um, 4096 × 3000 pixels) has been utilized. The MCP plate and phosphor bias voltages are set to be constant 1.75 kV and 5 kV, respectively. The number of channels in the X-ray radiation area on the MCP is approximately 10^6^, and the corresponding CMOS pixel number is 2 × 10^6^. Assuming the detection efficiency of hard X-rays on KBr coated MCP is ~ 1%, the detection scheme is nearly at the threshold of a single counting mode. The distance between ICS IP and the screen is 1.8 m and there are two Be window with thickness of 250 µm in front of the MCP assembly. The electron beam is fully deflected by a 90 deg electro magnet dipole located in front of the hard X-ray optics.

## Data Availability

Raw numerical data shall be extracted from each figure, and the color map in grayscale is linearly proportional to the intensity. Therefore, in principle, all data is contained in this report.
